# Inflammation due to ocular surface homeostasis imbalance caused by pterygia: tear lymphotoxin-alpha study and a literature review

**DOI:** 10.1186/s12348-024-00413-1

**Published:** 2024-06-14

**Authors:** Xie Fang, Guoli Lan, Yuan Lin, Zhiwen Xie, Yanlin Zhong, Shunrong Luo, Xianwen Xiao, Lianghuan Luo, Yiqiu Zhang, Hanqiao Li, Huping Wu

**Affiliations:** 1 Xiamen Eye Center and Eye Institute of Xiamen University, Xiamen, China; 2 Xiamen Clinical Research Center for Eye Diseases, Xiamen, Fujian China; 3 Xiamen Key Laboratory of Ophthalmology, Xiamen, Fujian China; 4 Fujian Key Laboratory of Corneal & Ocular Surface Diseases, Xiamen, Fujian China; 5 Xiamen Key Laboratory of Corneal & Ocular Surface Diseases, Xiamen, Fujian China; 6 Translational Medicine Institute of Xiamen Eye Center of Xiamen University, Xiamen, Fujian China

**Keywords:** Pterygium, Dry eyes, Tear film instability, Ocular surface homeostasis, Lymphotoxin-alpha

## Abstract

**Objective:**

To estimate the pterygium ocular surface state, and compare with healthy eyes and dry eyes. To investigate the inflammation due to pterygia growth by tear Lymphotoxin-alpha (LT α) test.

**Design:**

Prospective, single-center study.

**Participants:**

400 patients, divided into 100 pterygium group, 100 mild dry eye group, 100 moderate dry eye group, and 100 age-and sex-matched normal controls.

**Methods:**

The non-invasive break-up time (NIBUT), tear meniscus height (TMH) test, corneal fluorescein staining (CFS), meibomian gland loss score (MGs), and lipid layer thickness (LLT) were evaluated in all patients. Pterygium status and ocular status in the pterygium group were collected. The tear LT α test was conducted in the pterygium patients group.

**Result:**

Pterygium can affect the ocular surface, leading to decreased tear film stability. The TMH, NIBUT, CFS, MGs, and lipid layer thickness can provide insights into this phenomenon. The presence of pterygium can change the structure and condition of the ocular surface. Tear LT α testing shows an abnormal decrease in LT α levels in pterygium patients. This indicates an immune-inflammation microenvironment that causes tissue repair deficiency.

**Conclusion:**

The dry eye triggered by the growth of pterygium may originate from the tear film instability due to pterygia. As an inflammatory index, LT α in the development of pterygium and the aggravation of dry eye patients can indicate that the ocular surface is in different inflammatory states. Future tear testing in LT α may be a potential indicator to assess the inflammatory status of the dry eye.

## Introduction

Pterygium is a prevalent eye condition characterized by the abnormal growth of conjunctival tissue onto the cornea. The root cause of this ailment is multifaceted and can be attributed to chronic inflammation and microtrauma [1]. It can result in persistent eye irritation and dryness in the eyes. The tear film is a critical defense mechanism against environmental damage like dryness, chemical factors, and UV irradiation, which is crucial in safeguarding the ocular surface. However, the proliferation of pterygium can interfere with tear distribution, resulting in dry eye disease and altered tear dynamics. Recent research has found similarities between hypertrophic pterygium, tear film dysfunction, and meibomian gland dysfunction, including symptoms such as dryness and irritation [2]. These findings suggest that pterygium may cause pressure on the conjunctiva, leading to the compression of the meibomian gland [3]. Existing reports suggest a noteworthy association between the size of pterygium and the stability of tear membranes, indicating a possible impact of pterygium on the integrity of tear membranes [4]. Therefore, this gland dysfunction may contribute to the dry eye disorder experienced by pterygium patients.

Lymphotoxin-α is a member of the TNF superfamily. It was discovered in 1985 and initially named TNF-β that produced by the immune cells [5]. When tissues experience infection, damage, or inflammation, immune cells such as macrophages and T cells release LT α [6]. LT α can combine with LT-β to form trimeric ligands, which bind to LT-β receptors and activate NF-KB and other pathways [7]. Due to the presence of LT α polymorphism, TNF-α levels increase, which is associated with ocular surface inflammation via one of the potential mechanisms of related inflammatory reactions [8]. Previous research suggests that LT α2 has pro-inflammatory effects on corneal cells in vitro [9]. Cornea-α1β2 and cornea-βR cells express LT α and LT-β, leading to the production of inflammatory cytokines and chemokines through direct cell interaction [10].

Our research aimed to investigate the ocular surface parameters of individuals with unbalanced ocular surface homeostasis due to pterygium development. We conducted a comparative analysis between the eyes of healthy individuals and those with mild to moderate dry eye disease to identify both qualitative and quantitative changes in the tear film. As a biomarker, the LT α of tears is easy to detect and can indicate the number and function of goblet cells. This, in turn, can indirectly reflect the quality of tear film mucin. Detecting LT α can also provide further explanation regarding the imbalance of ocular surface homeostasis caused by pterygium inflammation. Our discoveries will help in understanding the relationship between structural ocular surface changes and the ocular surface status in patients with pterygium.

## Methods

The experimental protocol was established according to the ethical guidelines of the Helsinki Declaration. The studies involving human participants were reviewed and approved by the Human Ethics Committee of Xiamen University affiliated Xiamen Eye Center (XMIYEZX-KY-2024-004). The patients/participants provided their written informed consent to participate in this study. A study carried out by the Xiamen Eye Center from June 2022 to June 2023 involved 400 participants, with 158 men and 242 women. Those who had undergone pterygium resection or had a history of ocular surface diseases causing dry eye syndrome were excluded from the study.

The participants were divided into four groups, including a healthy control group, pterygium group and two groups with varying degrees of dry eye syndrome: Mild DED: just have some dry eye symptoms, such as eye foreign body sensation, dryness, redness, but no obvious damage to the eye surface. When staining the cornea, less than 5 dots; Moderate DED: ocular surface damage, but after treatment, the eye surface can return to normal. When staining the cornea, more than or equal to 5 less than 30 dots [11]. The ophthalmologist evaluated the patients using several non-invasive methods, including Non-invasive tear break up time (NIBUT), Tear meniscus height (TMH), meibomian gland loss (MGs), and lipid layer separation. The patients were also photographed using a slit-lamp camera to measure the size and thickness of pterygium. All tests were conducted and repeated between two graders ophthalmologist in one examination room, and patients were asked about their history of dry eye symptoms.

### TMH

The TMH was evaluated in a dark room using a corneal topography 5 M corneal camera (Oculus GmbH, Germany). The patient was asked to focus on the fixed target and project a disc of 22 rings onto the corneal surface. Pictures of the lower tear film meniscus were collected after 5s of blinking and TMH values were measured with an integrated ruler.

### NIBUT

During the NIBUT evaluation, the patient faced a 5 M corneal topographer with the chin supported under proper support. Then, the dorsal disc containing 22 red concentric circles was projected onto the patient’s eye, and the patient was asked to blink twice while staring at the center point. When the eyes remained open, the NIBUT value was determined and displayed on the screen with appropriate details related to the size of the tear film break up.

### MGs

The Keratograph 5 M ocular surface analyzer (OCULUS Germany): 0 points = no loss; 1 score = less than 1 / 3 of the total amount of meibomian glands; 2 points = 1 / 3 to 2 / 3 of the total amount of glands; 3 score = more than 2 / 3 of the total amount of meibomian glands. Each eye was scored between 0 and 4, and was scored for both eyelids [12].

### Lipid layer thickness (LLT) grading

Mean LLT measurements were obtained using a lipid view interferometer (Tears Science, Inc., Morrisville, North Carolina, USA). Briefly, the patient was instructed to remain fixed to the camera, which recorded a 20-second video of a tear film interferometry image. The unit of measurement used is the interferometric color unit (ICU), an index of the LLT corresponding to about 1 nm with an ICU [13]. With LLT greater than 100 nm, the laser map interferometer showed a maximum of 100 nm, followed by a lipid layer thickness score of 60 = 0; 60 ∼ 100 = 1; 100 + = 2.

### Corneal fluorescein staining(CFS)

Corneal fluorescein staining was performed 3 min after fluorescein infusion, and CFS was evaluated by a slit-lamp microscope illuminated with cobalt blue, in order to evaluate localised areas of corneal and conjunctival epithelial desiccation. Staining was recorded using the modified Oxford grading scheme [14].

### Assessment of the pterygium

The pterygium size, diameter, and congestion was assessed using a Haag-Streit BQ 900 slit lamp. Size: Grade I = 1: the head of the pterygium is at the edge of the cornea, and the conjunctiva of the conjunctiva is hypertrophic, with wing neovascularization. Grade II = 2: invasion of the pterygium head between the limbus and the pupillary margin; Grade III = 3: near the pupillary margin of the pterygium head; Grade IV = 4: invasion of the pterygium head or has crossed the pupillary area. The diameter of pterygium was defined as the margin from the limbus to the pterygium [15]. The congestion score: 0: the body of pterygium such as thin film, no obvious congestion 1: body such as thin film, mild congestion, light red color, slender blood vessels 2: body hypertrophy, red color, mild dilated blood vessels 3: body hypertrophy, dark red color, blood vessels significantly dilated.

### LT α

Patients used a disposable capillary tear collector to collect one drop of tears from the pterygium eye, according to their complaint. The collected tears were then dropped into the sample area of the LT α test card (Guangdong Shengze Kanghua Biomedical Co., LTD. ). After that, the LT α test reagent was added to the reagent area of the test card, and the name of the volunteer and the time of sampling were recorded on the card. The card was left for 10 min, and then it was placed in the card analyzer to read the parameters, including the qualitative results and LT α concentration. The immunochromatography process showed the qualitative results and LT α concentration at the end of testing. A red band was formed to determine if the chromatography process was as expected, regardless of whether the antigen was present in the sample or not.

### Statistical analysis

The database was collected and established using Excel software, and the statistical analysis was performed using SPSS 25.0 statistical software (SPSS Inc., Chicago 2017, USA). Continuous variables meet the normal distribution and equal variance is represented by the mean ± standard deviation (SD). Frequency and percentage representation of categorical variables and interpreted by correlation test. The ocular surface test assessed differences between groups (One-Way ANOVA test and post hoc test (LSD)).

## Result

The research involved a total of 400 patients who were split into three different groups. Group 1 consisted of 100 individuals who had healthy eyes, Group 2 included 100 patients with mild dry eye, and Group 3 had 100 patients with moderate dry eye. More detailed information about each group is available in Table 1. The findings revealed that the dry eye indicators varied between different groups, as demonstrated through Tear Meniscus Height (TMH) (*p* < 0.001; Table 1), Tear Break-Up Time (TBUT) (*p* < 0.001; Table 1), and CFS (*p* < 0.001; Table 1).

**Table 1:** General information of patients, examination, and test results(One-Way ANOVA test)

	Control group	Mild DED	Moderate DED	Pterygium	*P* Value
Sex					*P* < 0.001
Male	50	52	30	26	
Female	50	48	70	74	
Age (years)	23.40 ± 2.53	23.58 ± 3.02	23.30 ± 2.55	55.91 ± 10.44	*P* < 0.001
TMH (mm)	0.32 ± 1.48	0.16 ± 0.04	0.13 ± 0.04	0.14 ± 0.04	*P* < 0.001
NIBUT (s)	16.64 ± 4.36	7.06 ± 3.53	6.16 ± 2.34	5.32 ± 3.21	*P* < 0.001
CFS (score)	0	0.16 ± 0.37	1.54 ± 0.59	1.67 ± 1.36	*P* < 0.001
MG (up)	0.71 ± 0.51	0.79 ± 0.51	1.06 ± 0.7	1.32 ± 0.76	*P* < 0.001
MG (down)	0.82 ± 0.68	0.98 ± 0.75	1.22 ± 0.83	0.94 ± 085	*P* < 0.001
LLT (grading)	1 ± 0.34	0.37 ± 0.54	0.55 ± 0.7	0.60 ± 0.49	*P* < 0.001

DED, dry eye disease; LLT, lipid layer thickness; NIBUT, non-invasive break-up time; CFS, Corneal fluorescein staining; MG, Meibomian gland

In the post hoc test (LSD), the CFS of the pterygium group was higher than that of the control group, mild dry eye group, and moderate dry eye group (*P* < 0.001, *P* < 0.001, *P* = 0.004, respectively); The MGs (up) of the pterygium group were higher than those of the control group, mild dry eye group, and moderate dry eye group (*P* < 0.001, *P* < 0.001, *P* = 0.015, significantly); The MGs (down) of the pterygium group were higher than those of the moderate dry eye group (*P* < 0.001), but there was no significant difference compared to the control group and mild dry eye group (*P* = 0.857 and *P* = 0.067, respectively); The NIBUT of the pterygium group was lower than that of the control group (*P* < 0.001), but there was no significant difference compared to the mild and moderate dry eye groups (*P* = 0.326 and *P* = 0.562, respectively); The TMH of the pterygium group was lower than that of the control group (*P* = 0.046), but there was no significant difference compared to the mild and moderate dry eye groups (*P* = 0.853 and *P* = 0.883, respectively); The LLT of the pterygium group was higher than that of the mild and moderate dry eye groups (*P* < 0.001 and *P* < 0.001, respectively), but there was no significant difference compared to the control group (*P* = 0.102).

In the LT α tear test, The pterygium Grade I was 2.15 ± 5.17 dg/mL, Grade II was 0.58 ± 0.81 dg/mL, Grade III was 0.09 ± 0.24 dg/mL, Grade IV was 0.05 ± 0.07 dg/mL. We compared the pterygium grade and found that LT α showed a decreased trend with higher grade. In the correlation analysis of LT α, LT α was positively associated with NIBUT (Table 2). Furthermore, LT α pterygium size, pterygium grade, CFS, conjunctival congestion score, and LLT were negatively correlated (Table 2). In addition, the different kinds of pterygium was 0.58 ± 1.43 d g/mL in inflamed and 1.56 ± 2.31dg/mL in atrophic, which showed a statistical difference between the two (*P* = 0.016).

**Table 2:** Correlation test between LT α and clinical trials in the pterygium group

	LT-a	
	R	*P*
Pterygia diameter (mm)	-0.493	*P* < 0.001
Pterygium grade	-0.536	*P* < 0.001
NIBUT (s)	0.225	*P* < 0.001
Congestion (score)	-0.282	*P* < 0.05
*C*FS (score)	-0.301	*P* < 0.05
LLT (mm)	-0.398	*P* < 0.001

LLT, lipid layer thickness; NIBUT, non-invasive break-up time; CFS, Corneal fluorescein staining; LT α, Lymphotoxin-alpha

## Discussion

Pterygium is a prevalent ocular disease characterized by the development of triangular mucosal tissue on the ocular surface. The intricate relationship between pterygium and dry eye disease has long been a topic of study. Pterygium can cause ocular irritation, photophobia, and impaired tear stability [16]. Pterygium formation destabilizes the ocular surface by changing tear film dynamics, damaging conjunctival blood vessels, and altering meibomian glands [17]. Our research has revealed that the severity of pterygium is closely linked to the duration of tear film disruption. It is possible that the angle of tear evaporation may not be the underlying cause of the dry eye symptoms resulting from pterygium growth. This association is thought to be a result of structural changes brought on by pterygium growth, which can lead to an imbalance in the ocular surface [18], including tear secretion is reduced, tear film stability is compromised, evaporation is increased, epithelial cells deteriorate, tear osmolality rises, and goblet cell density decreases [19]. In this study, we showed a comprehensive evaluation of ocular surface parameters and structural integrity impacted by pterygium.

In healthy eyes, the conjunctiva and cornea maintain a stable relationship in both structure and function. However, the development of pterygium causes the conjunctival tissue to grow and extend onto the surface of the cornea, resulting in instability. This abnormal growth can cause eye irritation, redness, and a foreign body sensation [20]. Furthermore, it may disrupt the natural distribution of tears, which can lead to an uneven tear film on the eye surface and impact the eye’s protective function. Consequently, the eye can become more vulnerable to external factors like dry air and ultraviolet radiation [21]. Research has indicated that corneal abnormalities may play a role in the tear film’s instability within the eye [22]. Patients diagnosed with pterygium are likely to suffer from dryness and ocular discomfort due to the disturbance in the homeostatic balance and uneven distribution of tears, as demonstrated in various studies [23]. This may lead to pterygium patients with similar symptoms to patients with dry eye, further increasing eye discomfort. In this study, we found that pterygium had worse dry eye indicators than healthy eyes. In addition, pterygium patients were more destructive to the cornea and meibomian glands, but it had a more abundant LLT content than in patients with mild and moderately dry eyes. Therefore, the dry eye symptoms caused by the ocular surface structure and homeostasis changes of pterygium may not originate from the excessive evaporation of tears.

**Fig. 1:**
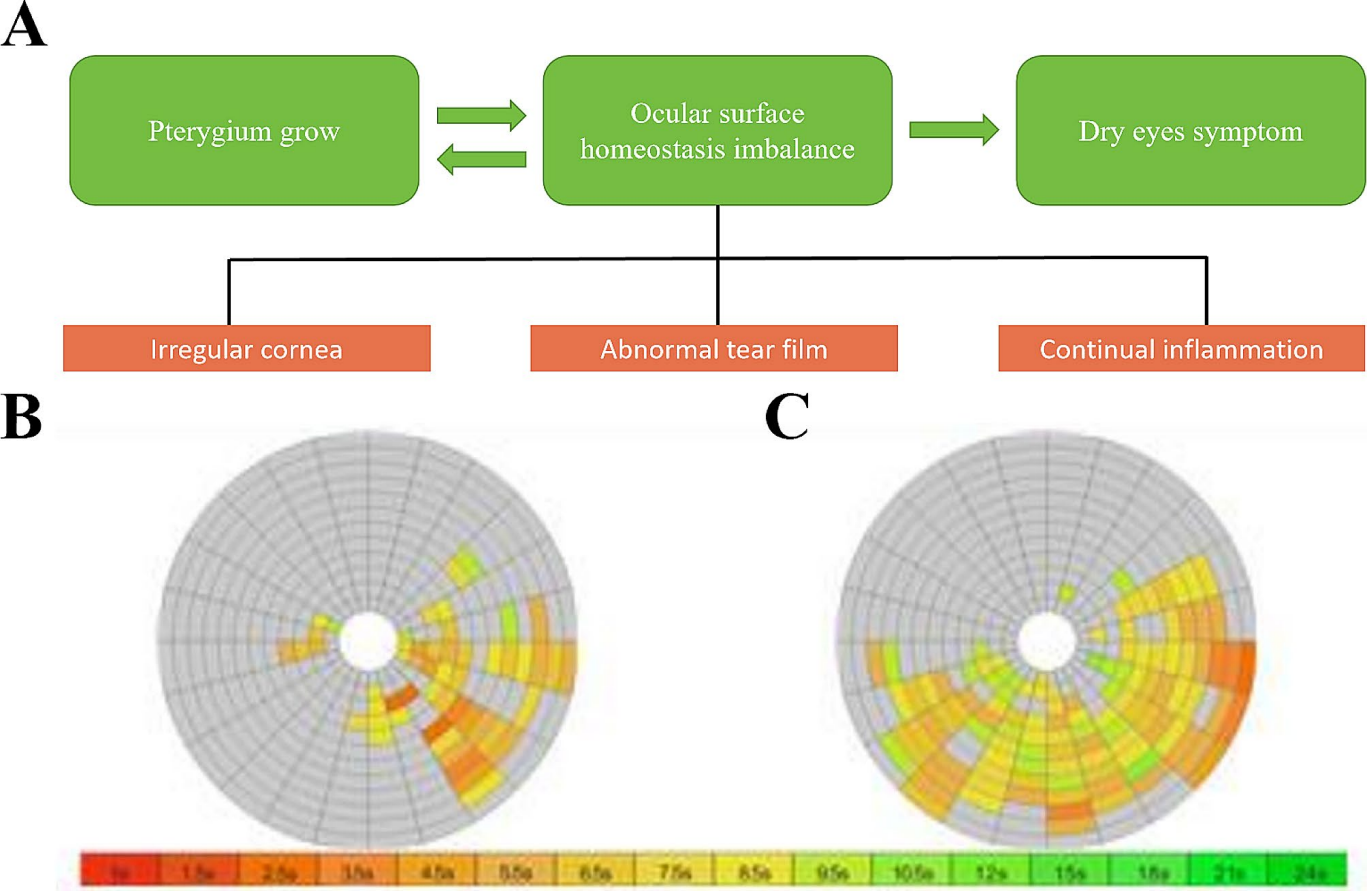
Relationship between pterygium and ocular surface homeostasis. (**A**) There may be an interactive relationship between pterygium growth and the imbalance of ocular surface homeostasis. When the irregular cornea, abnormal tear membrane and continuous inflammation may cause the growth of pterygium and the destruction of the ocular surface may lead to dry eye symptoms; (**B** and **C**) In the examination of tear membrane rupture, **B** shows abnormal tear break up time in the growth area, while **C** shows the lower half

Studies have suggested that there may be several reasons for the shortening of tear breakup time, including impaired blink pattern, epithelial drying and subsequent reduction in NIBUT, and irregularity of the ocular surface epithelium, which disrupts tear film stability by impairing surface tension and stability [24]. It is crucial to consider other factors such as hyperpermeability, inflammation, and instability in the structure of the ocular surface in the pathogenesis of ocular surface imbalance caused by pterygium [25]. Previous studies have shown that pterygium is characterized by marked vascular responses and marked inflammatory infiltrates [26]. Pterygium can cause inflammation and significant damage to the corneal epithelial surface and conjunctiva. During an active phase, it may lead to changes in the ocular surface and tear film abnormalities when compared to eyes without pterygium (Fig. 1). Our observations suggest that pterygium growth can cause corneal conjunctival defects, changes in vascularity, and congestion, and the inflammation that causes these changes is often overlooked as a risk factor for pterygium.

Research has indicated that UV radiation can result in genetic alterations impacting the cytokines IL-6 and IL-8 expression in individuals with pterygium [27]. These cytokines promote the production of MMPs and are commonly detected in the anterior portion of the pterygium. The discharge of IL-6, IL-8, and MMPs into the tear film may cause harm to the ocular surface [28], resulting in instability of the tear film, loss of epithelial cells, lowered mucus secretion, and enhanced tear permeability [29]. Patients with pterygium will have disturbances in tear film quality and quantity, and the reduction of conjunctival goblet cells may lead to tear film instability. Studies have shown that the pterygium resection can significantly increase the average goblet cell density and increase the secretion of mucin in the tear film [30]. Therefore, we believe that tear hypertonicity and tear membrane dysfunction are associated with pterygium and influence each other through certain mechanisms, ultimately leading to the ocular surface homeostasis imbalance.

LT α is a protein complex composed of three identical LT α subunits that mainly act on regulatory T cells (Treg). Treg and protein-mediating factors are essential for maintaining immune balance [31]. Treg suppresses the immune system and promotes tissue repair. Tregs have been shown to play a beneficial role in tissue repair, goblet cell differentiation, mucin secretion, and suppression of Th1 and Th17 cell proliferation. LT α helps maintain ocular surface immune balance through the LT α tumor necrosis factor receptor 2 Treg axis [32–34]. Our study revealed the state of LT α in pterygium, indicating the inflammatory activity of pterygium. Based on the measurement of LT α in ocular surface tear film, we found that the concentration of LT α in tears of patients with pterygium decreased. The absence and decrease of LT α reflect a decrease in cell proliferation and tissue repair ability, a decrease in the number and function of goblet cells, and a decrease in mucin secretion [35]. When there is an immune imbalance, mucus secretion and goblet cells decrease, leading to apoptosis and shedding. Additionally, Th17 cells secrete IL-17, which promotes the secretion of various vascular endothelial growth factors, resulting in corneal lymphangiogenesis [36]. These immune responses involve both the ocular mucosa and the systemic immune response. squamous metaplasia of ocular surface epithelial cells caused by immune imbalance may be responsible for the active state [37]. This leads to immune cell entry into the ocular surface, which hinders Treg activity, further expanding and migrating Th17 and Th1 cells and exacerbating epithelial damage [38].

**Fig. 2:**
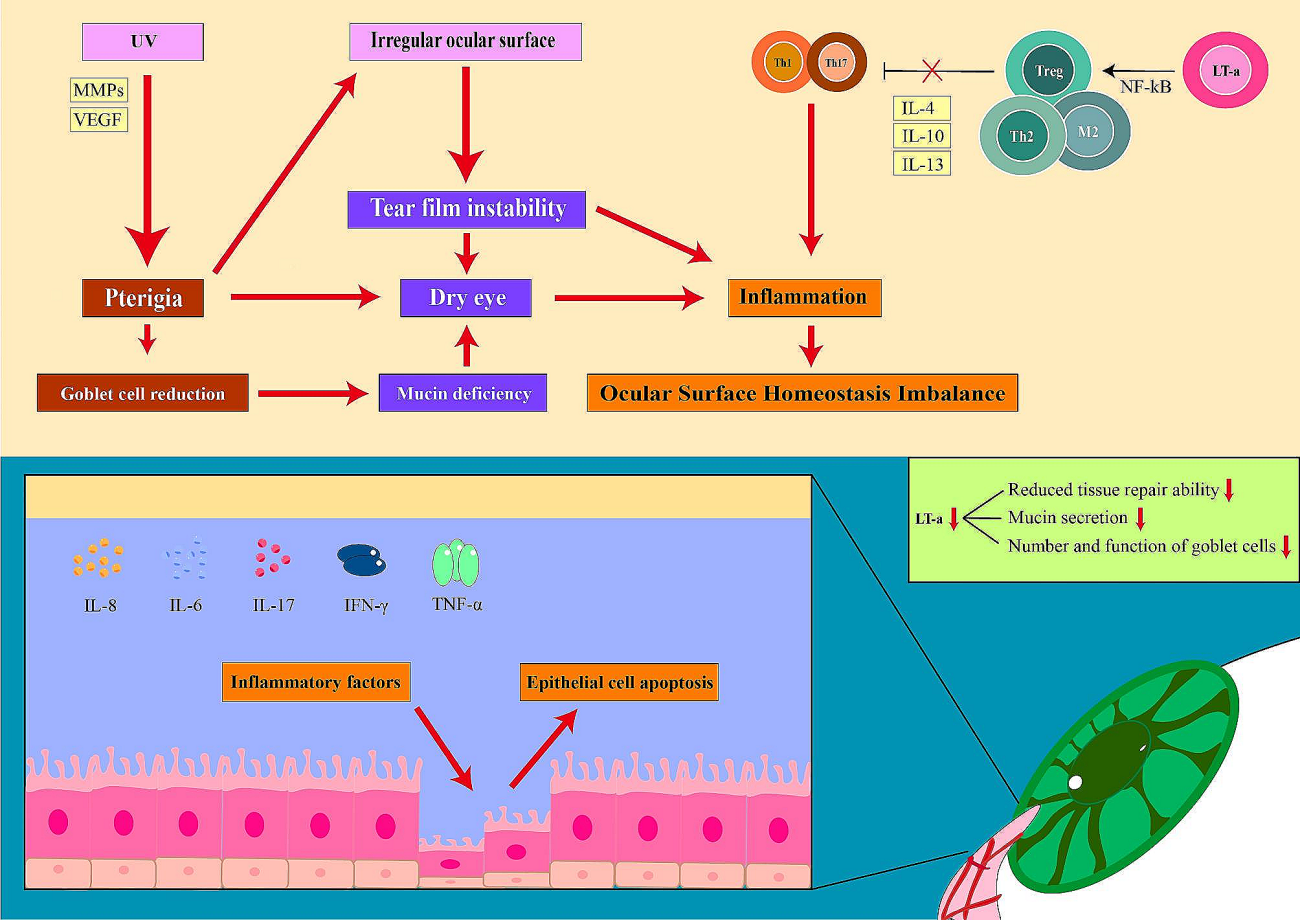
Pterygium causes an inflammatory reaction and LT α may serve as one of the inflammatory factors. Repetitive inflammation involved with ultraviolet (UV) combined with matrix metalloproteinases (MMPs) and vascular endothelial growth factor (VEGF) acts at the conjunctiva can cause the formation of pterygium. This abnormal growth triggers the release of inflammatory substances like IL-6, IL-8,IL-17, interferon gamma (IFN-γ) and tumor necrosis factor alpha (TNF-α) into the tears, causing an inflammatory reaction that destabilizes the tear film. Additionally, lower levels of LT α indicate impaired tissue repair on the eye’s surface. As pterygium advances, the reduction in goblet cells and tear mucin may be linked to the decreased LT α which The reduced ability of the activation of NF-kB leading to an impaired anti-inflammatory response from Treg / Th and / or M2 to reduce the IL-4, IL-10 as well as the immune regulatory function of IL-13 which to inhibit Th 1 and Th 17 leads to inflammation.This progression leads to a harmful cycle of immune inflammation and chronic irritation, ultimately disrupting the ocular surface balance

Immune cells such as macrophages, goblet cells in ocular surface tissue, and Tregs secrete important growth factors, cytokines, immune tolerance factors, antimicrobial enzymes, peptides, and mucins that are crucial for maintaining normal conjunctival epithelial tissue proliferation, differentiation, and function [39]. These factors are essential for ocular surface homeostasis, epithelial tissue density, repair, and other normal functions. Therefore, maintaining tear film quality, immune tolerance, and tissue repair are critical for maintaining eye surface immune homeostasis. In order to gain a complete understanding of the relationship between pterygium-induced structural changes in ocular surface structure and ocular surface homeostasis, additional histopathological studies are required. This study evaluated the clinical parameters of the ocular surface in individuals with pterygium and examined the correlation between pterygium status and ocular surface homeostasis (Fig. 2). Our findings indicate that changes in the ocular surface structure can compromise ocular surface homeostasis, and the “irregular” ocular surface resulting from pterygium warrants further investigation. This study compared the decrease of related dry eye indicators caused by pterygium with those of dry eye patients without pterygium. The limitation of this study is the need to further explore a confounding factor in the dry eye cohort in future studies, requiring further validation in more prospective studies. These results contribute to our understanding of the disease and provide a fresh perspective for future research.

## Conclusion

The ocular surface changes of pterygium caused non-evaporative DED. Detection of LT α suggested the dry eye, pterygium and the relationship with inflammation. The inflammation of the conjunctival tissue may lead to insufficient tear secretion in pterygium patients. It is valuable To further explore the relationship between the lymphatic toxin family and the pathogenesis of pterygium as well as ocular surface inflammation reactions.

## Data Availability

The data presented in this study are included in the article. The data are not publicly available due to restrictions that apply to the availability of the data (e.g., privacy or ethical). Datasets from this study may be available upon request from the corresponding author and provided upon approval from the sponsor and in accordance with data privacy and ethical provisions.

## Abbreviations

LT α: Lymphotoxin-alpha
DED: Dry eye disease
LLT: Lipid layer thickness
NIBUT: Non-invasive break-up time
TMH: Tear meniscus height
CFS: Corneal fluorescein staining
MGs: Meibomian gland loss score
Treg: Regulatory T cells
